# Stage-related transcriptional changes in HR-positive/HER2-negative breast cancer

**DOI:** 10.1038/s41523-026-00973-6

**Published:** 2026-07-02

**Authors:** William S. O. Symmans, Kevin Tran, Chunxiao Fu, Hongxia Sun, Rebekah Gould, Lajos Pusztai, Tari King, Roberto Salgado, Vicente Valero, W. Fraser Symmans, Eleni Andreopoulou

**Affiliations:** 1 The University of Texas MD Anderson Cancer Center, Houston, TX USA; 2 Yale University, New Haven, CT USA; 3 Winship Cancer Institute, Emory University, Atlanta, GA USA; 4 ZAS Hospitals, Antwerp, Belgium; 5 Weill Cornell Medicine, New York City, NY USA

**Keywords:** Biomarkers, Cancer, Computational biology and bioinformatics, Oncology

## Abstract

It is important to understand how the biology of breast cancer might differ with the burden of disease. Analyzing the expression of cancer-relevant genes with increasing diagnostic stage can provide insight into the changing biology of breast cancer and may also be clinically useful. Here, we evaluated the transcriptional activity according to the stage of breast cancer (American Joint Committee on Cancer, AJCC Stage I to IV) from 1152 patients with hormone receptor-positive HER2-negative breast cancer. Fresh tumor samples were prospectively collected for clinical-translational research with transcriptional profiling. Most transcriptional signatures remained consistent across stage categories, but the significant changes associated with increasing stage were attributed to endocrine escape, decreasing differentiation, increased complexity of signal transduction, and a few metabolic changes. The clinical relevance and implications of these findings are discussed. The expression data from these high-quality samples are a new publicly available resource for researchers.

## Introduction

Staging of breast cancer using the American Joint Committee on Cancer (AJCC) system is an important measure of the extent and risk of the disease^1^. Consequently, staging is an essential tool used for planning the course of treatment and making adjustments based on the estimated level of risk. However, a biological assessment that reflects the stage and state of disease is also critical for understanding the full spectrum of a patient’s disease and for developing a personalized therapeutic plan. For example, gene expression profiles have greatly impacted treatment approaches for patients with Stage I-IIA hormone receptor-positive, HER2-negative (HR+/HER2−) cancer^2–4^. There is also potential to apply gene expression profiles to more advanced stages of disease to guide treatment. In this context, a comparison of static and dynamic changes in gene expression profiles across the stage categories of HR+/HER2− breast cancer can provide a useful perspective on which clinically or biologically relevant profiles can differ by stage of disease in a population.

Our approach was to compare populations defined by stage categories to test for associations between stage of disease and expression level of single genes, multigene prognostic or predictive signatures with established relevance to breast cancer, and more generic cancer biology signatures, many of which relate to the 14 established Hallmarks of Cancer^5^. We assembled a large cohort of fresh clinical samples that were prospectively collected on research protocols and optimally preserved for high-quality RNA^6–9^. All had gene expression profiling performed using whole transcriptome microarrays.

Adaptation of clinical sampling methods for research sample procurement to different clinical scenarios allowed us to successfully obtain cancer samples for different research protocols. That is because fine needle aspiration (FNA) cytology is generally well accepted for research collection when there is advanced disease, or when diagnosis has already been established, and the patient will proceed with neoadjuvant treatment^6^. In those scenarios, there may be no need for another core biopsy for research. Otherwise, the prospective collection of fresh tissue samples is more acceptable when patients have been diagnosed with invasive breast cancer, and their primary treatment is surgery. Thus, fresh tumor prospective research samples may come from FNA cytology, core biopsy tissue, or fresh breast cancer tissue collected at the time of surgery, and the type of sample is influenced by the stage of the disease and clinical logistics. This also introduces a major preanalytical effect due to differences in the cellular populations within cytology and tissue samples^10^. Therefore, we formally compared matched pairs of cytology and tissue samples that were collected in a subset of patients at the time of surgery, specifically to calibrate gene expression measurements between cytology and tissue samples^8^. This comparison enabled us to account for the type of clinical sample when interpreting differences in gene expression levels as attributable to differences by stage of disease.

Overall, this study examines stage-associated changes in the expression of single genes and gene expression signatures of proven relevance to patients with HR+/HER2− breast cancer, along with gene expression profiles that represent important biological signatures of cancer. Notably, we studied this in high-quality samples that were collected prospectively in clinical research protocols and accounted for whether those were cytology or tissue samples. Finally, we present a novel cohort of prospectively collected, high-quality RNA expression profiles from clinical research protocols, which is a new data resource for others to study for stage-related changes in HR+ breast cancer.

## Results

### Patient and sample characteristics

**Fig. 1:**
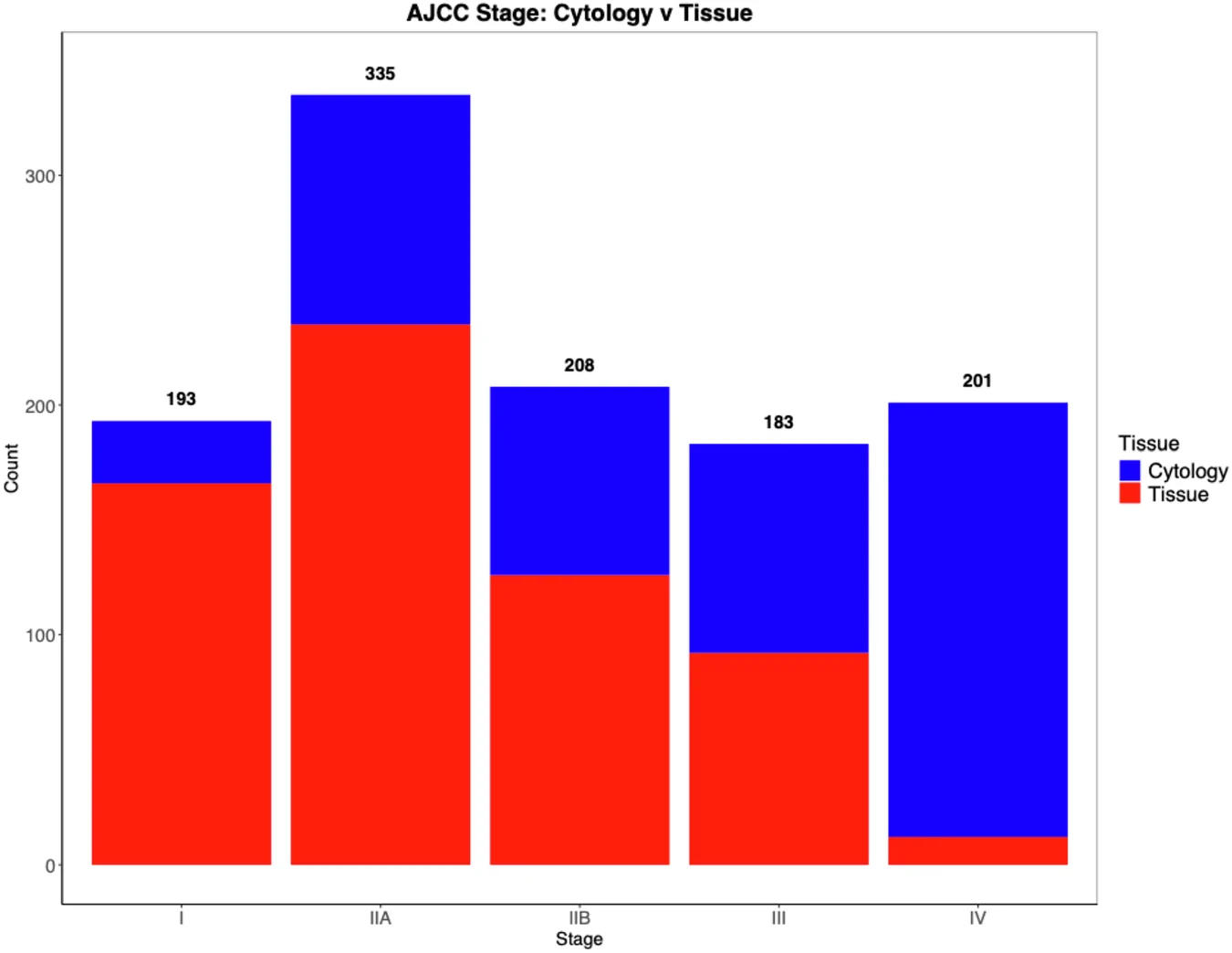
The number and type of specimens collected from patients at each AJCC stage. The red bars represent patients from whom cytology samples were collected, and the blue bars represent the patients from whom tissue samples were collected.

The study cohort consisted of 1152 HR+/HER2− breast cancers (see REMARK diagram in Supplementary Fig. S1). There was sufficient representation of samples across five stage categories (I, IIA, IIB, III, IV), although a greater number of samples were from Stage IIA (Fig. 1). Tissue samples were more commonly collected from patients with lower stage disease (Stages I and IIA) and were usually from surgically resected breast cancer. Cytology samples were more frequently collected from patients with more advanced stages, particularly Stages III and IV (Fig. 1). The cohort included matched pairs of cytology and tissue samples collected at the time of surgery from 75 patients.

### Differences across the spectrum of Stage I to Stage IV

Table 1 shows the results from the selected transcripts and multigene signatures that had significant regression of expression values as the stage increased (*p* < 0.001). All other results are shown in Supplementary Table S1.

**Table 1:** Breast cancer-specific genes and clinical signatures of interest

Signature	Sample matched pairs			Clinical samples cohort					
	Cytology vs tissue			Cytology vs tissue			AJCC stage effect		
	Estimate	95% CI	*p* value	Estimate	95% CI	*p* value	Estimate	95% CI	*p* value
Single genes (probesets)									
* ESR1* Probeset	0.311	−0.121, 0.743	0.157	−0.233	−0.415, −0.051	0.012	−0.121	−0.188, −0.055	**<0.001**
* PGR* Probeset	0.096	−0.088, 0.280	0.302	0.025	−0.049, 0.098	0.512	−0.058	−0.084, −0.031	**<0.001**
* ERBB2* Probeset	0.279	0.072, 0.487	**0.009**	−0.154	−0.257, −0.051	**0.003**	−0.056	−0.094, −0.019	**0.003**
* AURKA* Probeset	0.109	−0.041, 0.259	0.153	0.184	0.109, 0.258	**<0.001**	0.063	0.036, 0.090	**<0.001**
Breast cancer multigene signatures									
OncotypeDX	−5.068	−14.496, 4.360	0.290	1.690	−0.958, 4.339	0.211	2.198	1.233, 3.162	**<0.001**
rorS	5.285	−3.262, 13.831	0.224	11.141	7.747, 14.536	**<0.001**	5.102	3.866, 6.338	**<0.001**
endoPredict	0.134	−0.651, 0.920	0.736	0.907	0.595, 1.220	**<0.001**	0.480	0.366, 0.594	**<0.001**
GGI	0.094	0.004, 0.184	0.042	0.132	0.097, 0.167	**<0.001**	0.033	0.020, 0.045	**<0.001**
SETERPR	0.092	−0.139, 0.322	0.434	−0.040	−0.126, 0.046	0.363	−0.104	−0.135, −0.073	**<0.001**
PIK3ges	0.108	−0.130, 0.346	0.371	−0.176	−0.267, −0.085	**<0.001**	−0.072	−0.105, −0.039	**<0.001**
Cancer biology signatures									
Hallmark_PI3K_AKT_MTOR_signaling	0.014	−0.033, 0.062	0.559	−0.022	−0.044, −0.000	0.045	0.014	0.006, 0.022	**0.001**
SRC Signature	−0.021	−0.040, −0.001	0.041	−0.022	−0.033, −0.012	**<0.001**	−0.009	−0.013, −0.005	**<0.001**
IMMUNE Signature	−1458.583	−1946.473, −970.693	**<0.001**	93.247	−149.601, 336.095	0.452	146.009	57.573, 234.444	**0.001**
CIN70 Signature	0.126	0.036, 0.216	**0.006**	0.111	0.075, 0.148	**<0.001**	0.025	0.012, 0.038	**<0.001**
HALLMARK_SPERMATOGENESIS	0.063	0.021, 0.105	**0.003**	0.182	0.162, 0.202	**<0.001**	0.014	0.007, 0.021	**<0.001**
HALLMARK_HEME_METABOLISM	−0.001	−0.034, 0.032	0.951	0.056	0.035, 0.078	**<0.001**	0.018	0.010, 0.026	**<0.001**

A cancer biology signature was included from the 50 listed in the Broad Institute Gene Set Enrichment Analysis if its stage effect was highly significant (*p* < 0.001). For the matched pairs cohort, this meant that cytology and tissue were significantly different. For the clinical samples cohort, this meant that the gradient of the regression line across staging was significantly up or down. The “estimate” column quantifies the difference in means between cytology and tissue for matched pair samples, with a positive value indicating that the cytology was higher than tissue. “Estimate” in the clinical cohort is the slope of the regression line with increasing stage category as an integer.

#### Single genes that discriminate breast cancer subsets

**Fig. 2:**
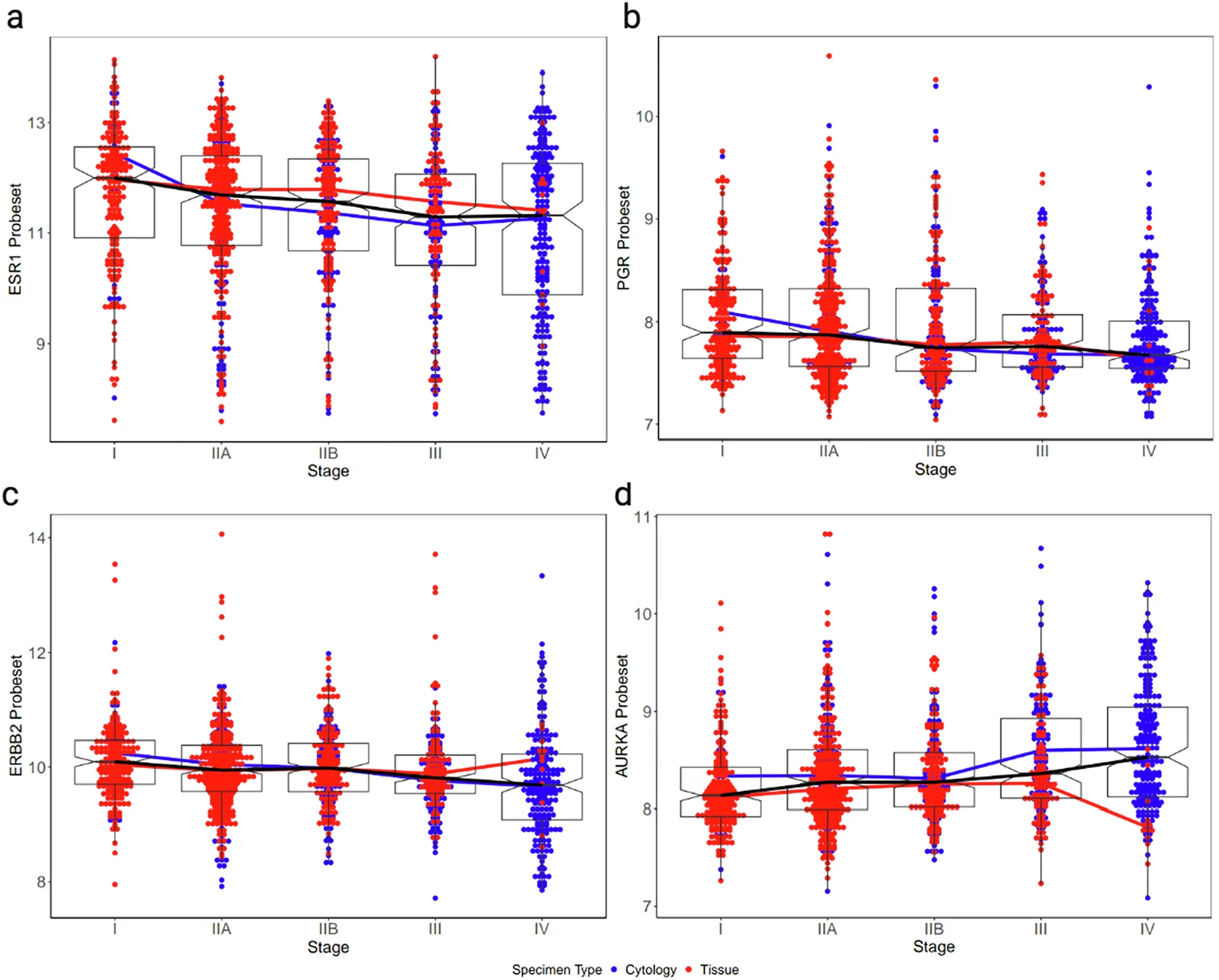
Box plots showing mean expression values and interquartile range for relevant single genes across the stage categories. **a** *ESR1*, **b** *PGR*, **c** *ERBB2*, and **d** *AURKA*. Each blue dot represents a patient from whom a cytology sample was collected, and the blue line shows the average expression of that sample group at each stage. The red dots and lines represent patient-specific and mean expression levels per stage for the tissue samples, respectively. The black line connects the mean values from all samples. The blue and red lines connect the mean values for cytology and tissue samples, respectively.

There was no significant difference in the expression of *ESR1*, *PGR*, or *AURKA* in the matched pairs of cytology and tissue samples (Table 1). Expression of *ERBB2* was significantly higher in cytology samples (Table 1). We observed a small, but statistically significant change in the expression levels of *ESR1*, *PGR*, and *ERBB2*, and a significant increase in the expression level of *AURKA* with increasing stage category (Table 1, Fig. 2).

#### Multigene signatures

**Fig. 3:**
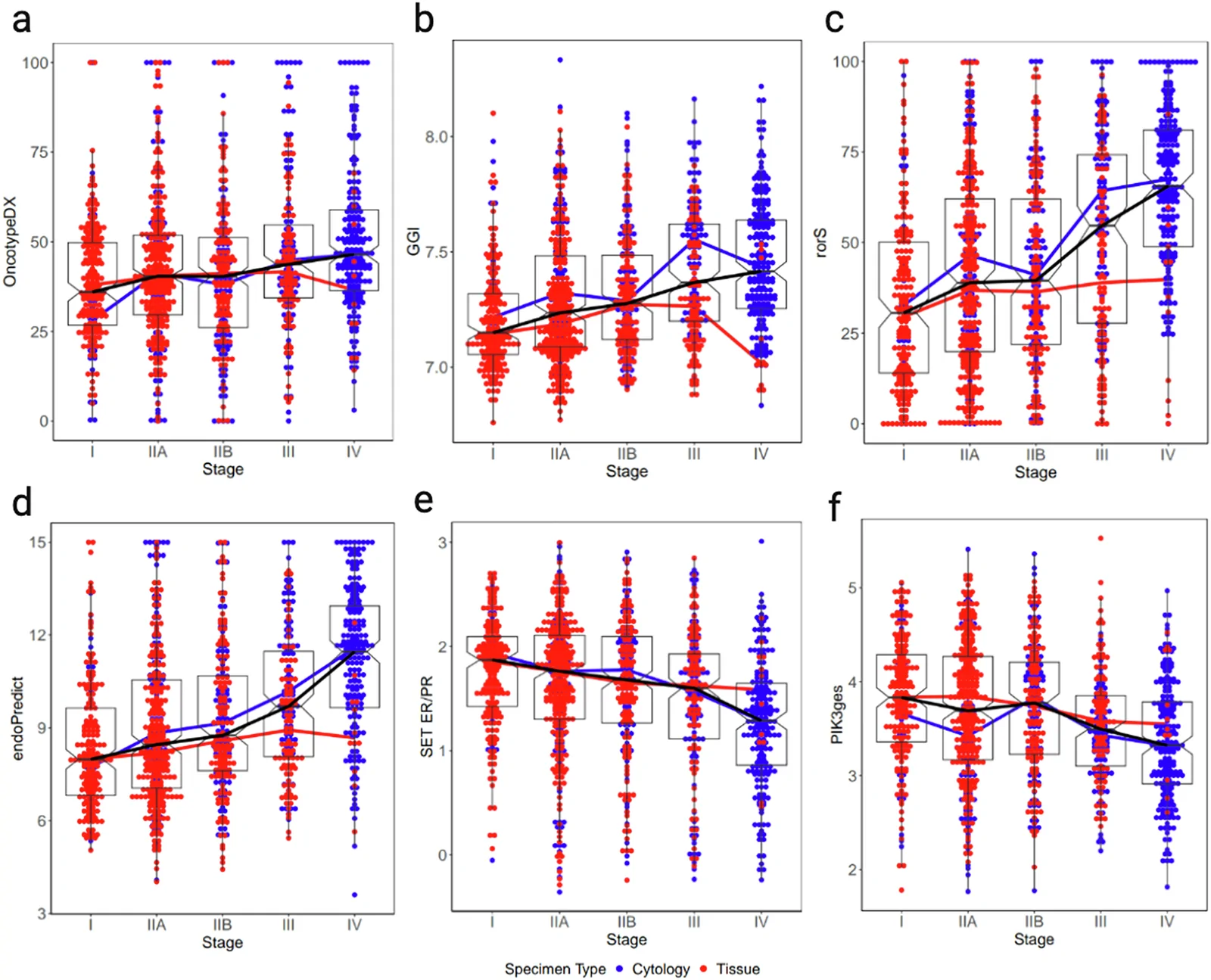
Box plots showing mean expression values and interquartile range for breast cancer multigene signatures across the stage categories. **a** Recurrence Score, **b** ROR-S, **c** EndoPredict, **d** GGI, **e** ${\mathrm{SET}}_{\mathrm{ER}/\mathrm{PR}}$ index of endocrine activity, and **f** PI3-kinase gene expression signature. Each blue dot represents a patient from whom a cytology sample was collected, and the blue line shows the average expression of that sample group at each stage. The red dots and lines represent patient-specific and mean expression levels per stage for the tissue samples, respectively. The black line connects the mean values from all samples. The blue and red lines connect the mean values for cytology and tissue samples, respectively.

GGI was the only multigene signature that differed by sample type in matched sample pairs, being slightly higher in cytology samples (Table 1). The expression levels of multigene prognostic signatures were significantly increased in higher stages of disease (Fig. 3). Specifically, Recurrence Score and GGI demonstrated consistent, incremental increases (Fig. 3a, b), whereas ROR-S and EndoPredict showed a gradual increase from Stage I to IIB, then a greater increase from Stages IIB through IV (Fig. 3c, d). On the other hand, there was a steady downward trend in the signature that quantifies endocrine transcriptional activity attributable to estrogen receptor and progesterone receptor, i.e., the sensitivity to endocrine therapy ${\mathrm{SET}}_{\mathrm{ER}/\mathrm{PR}}$ index (Fig. 3e). There was the same downward trend with a signature that represents gene expression related to activating mutation of the *PIK3CA* gene in HR+ breast cancers and represents overactivity of PI3-kinase mediated signal transduction (i.e., PIK3ges). The expression levels of PIK3ges had a relatively flat trend from Stage I to IIB (Fig. 3f). The type of tumor sample contributed independently to the stage-related trends for every multigene signature, except for Recurrence Score and ${\mathrm{SET}}_{\mathrm{ER}/\mathrm{PR}}$ index (Table 1).

#### General biological signatures attributable to cancer

**Fig. 4:**
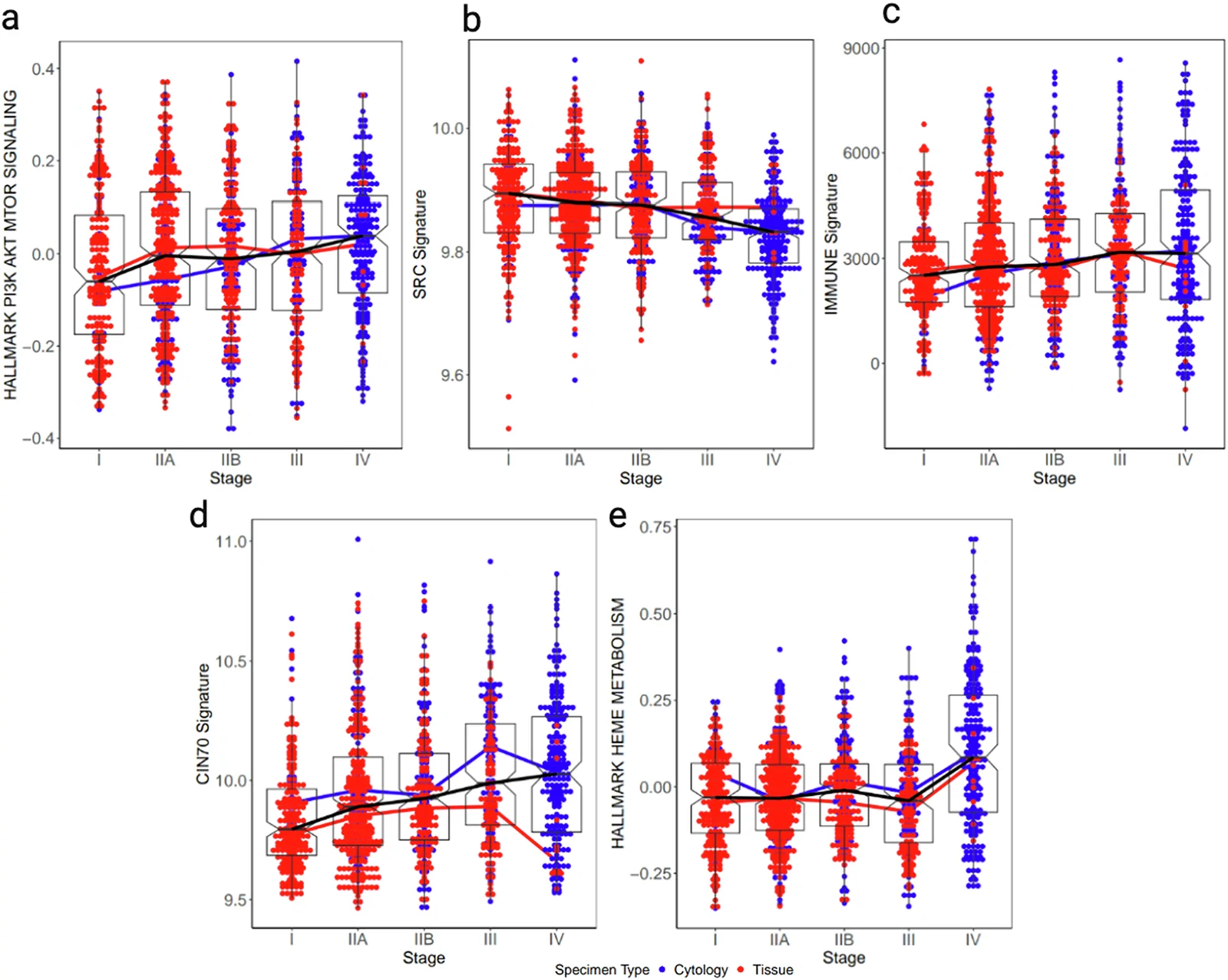
Box plots showing mean expression values and interquartile range for noteworthy signatures related to breast cancer or generic cancer biology across the stage categories. **a** PI3K-AKT-MTOR Signaling, **b** SRC Signature, **c** Immune Signature, **d** CIN70, and **e** Heme Metabolism. Each blue dot represents a patient from whom a cytology sample was collected, and the blue line shows the average expression of that sample group at each stage. The red dots and lines represent patient-specific and mean expression levels per stage for the tissue samples, respectively. The black line connects the mean values from all samples. The blue and red lines connect the mean values for cytology and tissue samples, respectively.

The expression levels of multigene signatures representing generic cancer biology were mostly consistent across the stages of breast cancer. Those with a highly significant stage-related change (*p* < 0.001) are shown in Table 1, the others in Supplementary Table S1. There was a slight increase in the expression of a cancer biology signature representing signal transduction through the PI3-kinase, AKT, and MTOR pathway (Fig. 4a), and a steady decrease in the expression of a multigene signature for signal transduction via SRC (Fig. 4b). The expression of a multigene signature representing immune activity within the tumor increased slightly as the stage increased (Fig. 4c). A signature for chromosomal instability increased steadily from Stages I to IV (Fig. 4d), as did a spermatogenesis signature representing the “stem-like” activation of cancer cells to promote proliferation (Table 1). Finally, a signature representing deregulated porphyrin-related heme metabolism remained flat from Stages I to III but increased significantly from III to IV (Fig. 4e, Table 1).

### Stage-related differences within Stage IV based on progression events

**Fig. 5:**
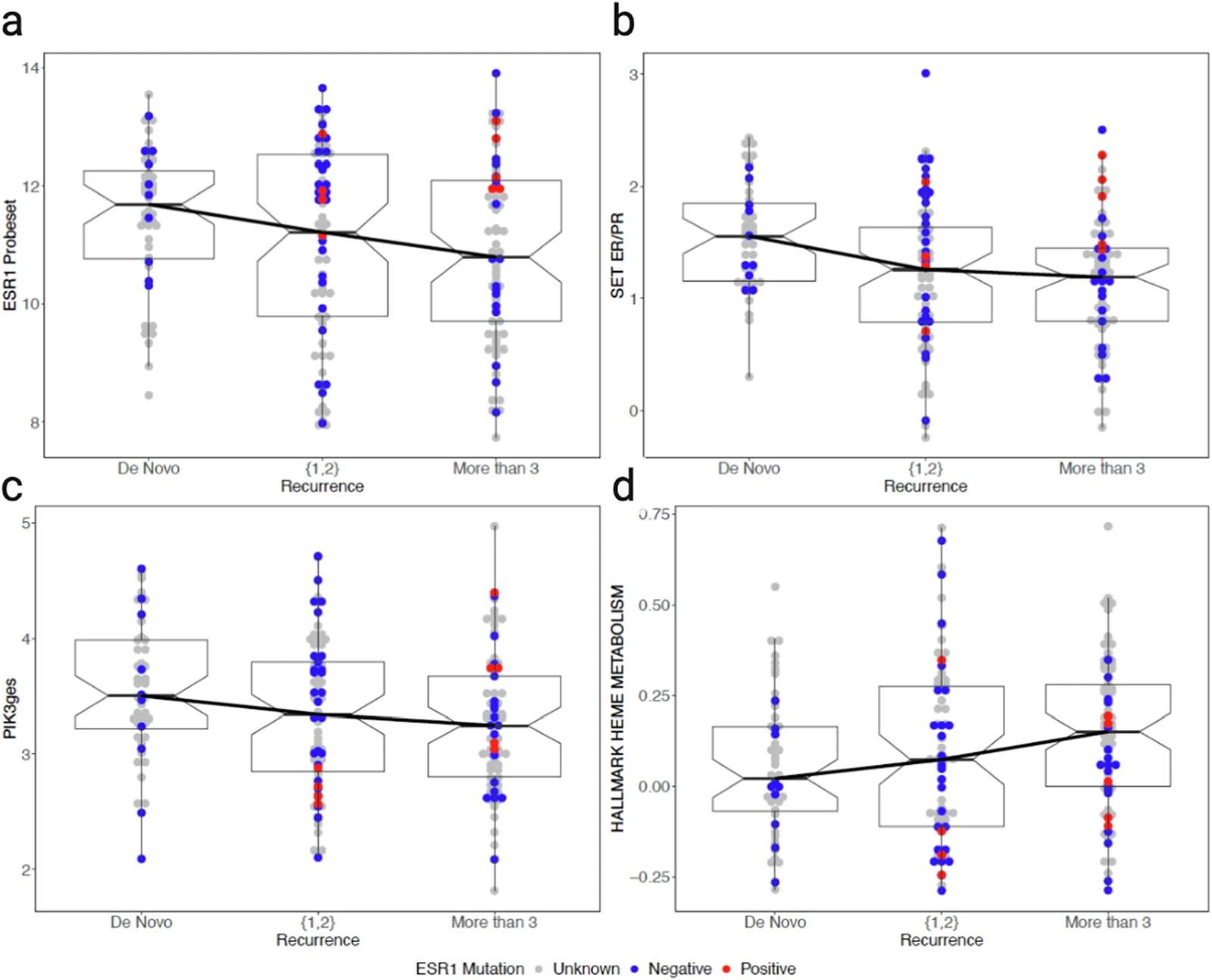
Box plots showing mean expression values and interquartile range for signatures related to breast cancer or generic cancer biology as the recurrence of disease increases. **a** ESR1 Probeset, **b** ${\mathrm{SET}}_{\mathrm{ER}/\mathrm{PR}}$ index of endocrine activity, **c** PI3-kinase gene expression signature, and **d** Heme Metabolism. Red dots indicate the presence of an *ESR1* mutation, blue dots indicate a lack of mutation, and gray dots indicate an unknown mutation status. The black line connects the mean values from all samples.

Figure 5 presents the most significant changes in single genes or multigene signatures within Stage IV cancer samples categorized by when they were collected: de novo, 1st or 2nd recurrence, or 3rd or later recurrence. The expression of the estrogen receptor gene was decreased in samples from later recurrence (Fig. 5a), as was the expression of the ${\mathrm{SET}}_{\mathrm{ER}/\mathrm{PR}}$ index of endocrine activity (Fig. 5b). Samples with a mutation of *ESR1* were from recurrent disease appeared to have higher expression levels of *ESR1* and ${\mathrm{SET}}_{\mathrm{ER}/\mathrm{PR}}$ index than samples without an *ESR1* mutation, although high expression was also seen in some metastases that did not have *ESR1* mutation. Average ${\mathrm{SET}}_{\mathrm{ER}/\mathrm{PR}}$ index expression declined on the first recurrence of breast cancer, then remained relatively stable in samples from later recurrences (Fig. 5b). The decrease in median expression level of PIK3ges had a similar pattern to ${\mathrm{SET}}_{\mathrm{ER}/\mathrm{PR}}$ index (Fig. 5b, c). There was a strong upward trend in the expression of genes representing deregulated cellular energetics through heme metabolism with an increasing number of recurrence events (Fig. 5d).

## Discussion

In our study, most transcriptional signatures did not change significantly, including several important biological signatures representing glycolysis, hypoxia, and apoptosis. However, certain biological themes of stage-related change were noteworthy. Generally, we observed a lower state of differentiation (including diminution of endocrine signaling), changes in signal transduction profiles, an increase in proliferation and genomic grade, increasing immune activity and instability of the genome, and some evidence of metabolic dysregulation in more advanced stages of HR+/HER2− breast cancer. A more detailed analysis of Stage IV cancers based on the number of progression events suggested a shifting away from endocrine signaling and PI3-kinase-mediated activity, with a shift towards some aspects of changing energy metabolism. Our findings point towards a modest overall change in the cellular functions within the cancer as it advances in stage. Ultimately, the treatment protocol adapts to the dynamic biological changes acquired through the course of the disease, eventually informing the potential transition to cytotoxic treatment as a late resort in an advanced HR+ setting.

We observed endocrine escape as decreasing SET_ER/PR_ index and *ESR1* with increasing stage. The former was consistent through Stages I to III, and from de novo through progressive Stage IV disease, i.e., through the entire continuum of stage and progression. We are aware that a recent longitudinal study of patient-matched HR+/HER2− breast tumors reported an increase in *ESR1* gene expression from early cancer to metastatic^11^. However, this does not necessarily conflict with our findings because that study measured gene expression only in samples from patients who developed metastatic cancer, whereas most of our patients would not have recurred. The cancers most susceptible to hormonal therapy are less likely to metastasize, and vice versa, possibly reflected by a limited emergence of acquired *ESR1* mutation. This difference in population may also explain why we observed a decrease in *ERBB2* expression, while they found an increase.

Our study enhances the perspective of the current evolving paradigm in clinical practice for close monitoring and early detection of recurrent disease in high-risk patients. Recent trials reported that early detection of *ESR1* mutation before radiographic progression could prompt a treatment change that improved progression-free survival in patients with early-stage disease^12^. There is now a validated benefit to closely monitoring high-risk disease in early stages, guided by the emergence of *ESR1* as a targetable genetic alteration^13^. *ESR1* mutations, captured in ctDNA, are likely an adaptive consequence of secondary resistance to Aromatase Inhibitors (AI)^14^. In this setting, treatment aims to address the dynamic evolution of the biological state of the disease, and longitudinal molecular characterization methods offer an opportunity for precision medicine; Selective Estrogen Receptor Degraders (SERDs) were proven more effective than the standard AI in treating HR+/HER2− breast cancer with mutated *ESR1*. Tracking *ESR1* mutation status in ctDNA is a sensitive way to monitor progression and diagnose early recurrence of endocrine-resistant disease.

After endocrine activity, the second most important driver in hormone receptor-positive breast cancer is signal transduction, particularly through the PI3-kinase pathways. *PIK3CA* is the most commonly mutated gene in HR+/HER2− breast cancer at a rate of 40%, so characterizing its expression across the stages gives insight into a key driver of the disease^15^. We observed a seemingly discordant decrease in the multigene PI3-kinase gene expression signature with increasing stage and number of recurrences and an increase in the collection of genes representing activating mutations of the PIK3-AKT-MTOR pathway. One explanation is that signaling complexity increases with stage, such that the cancer might not be as reliant on *PIK3CA* activity, specifically as alternative uses of this signal transduction mechanism evolve^16^. In other words, the early biology of PI3-kinase seems to be replaced by other signal transduction opportunities as the cancer progresses. However, the status of *PIK3CA* mutation in primary and metastatic breast cancer has been shown to be largely concordant (69%)^17^. Another possible explanation is that an upstream activator of the pathway overpowers the declining PI3-kinase activity, thereby increasing the expression of AKT and MTOR. Or, despite diminished PI3-kinase activity, a repressor of downstream activity is removed.

A different measure of signal transduction in cancer changed in the opposite direction to PI3K-AKT-MTOR across the stages. SRC is an upstream activator of PI3K-AKT-MTOR signaling and other signaling pathways^18^. The balance of these two signatures suggested a subtle shift towards PI3K-AKT-MTOR signaling in advancing stages of cancer and away from the upstream SRC pathway that also promotes cell growth and survival. In addition, the expression of the *ERBB2* gene for HER2 declined with advancing stage. Overall, within these trends, it seemed that membrane signaling remained consistent across Stages I to IIB, then dropped off slightly in Stages III and IV.

The presence of a *PIK3CA* mutation is addressed in clinical studies that investigate primary resistance to endocrine therapy. Recently, Inavolisib (PI3Kα inhibitor) plus Palbociclib (CDK4/6 inhibitor) and Fulvestrant (SERD) was the first FDA-approved triplet combination treatment used as a first-line treatment for patients with *PIK3CA*-mutated, locally advanced or metastatic HR+ breast cancer. These patients were considered to have endocrine-resistant disease after recurrence on or after completing adjuvant endocrine therapy^19^. Additionally, the CAPItello-291 trial investigated the combination of Capivasertib (AKT inhibitor) and Fulvestrant in a population that progressed after AI treatment with or without CDK4/6 inhibitor^20^. They demonstrated a progression-free survival benefit independent of both the CDK4/6 inhibitor exposure and the detection of AKT pathway alteration.

When HR+/HER2− breast cancer does progress, it seemingly becomes less differentiated. Overall, we observed that the multigene expression signatures that represent lesser differentiation were increased with advancing stage. With ROR-S and EndoPredict, but not Recurrence Score, this change was most pronounced at later stages, which fits into the concept that the expression of genes associated with a diminished state of differentiation should reflect more aggressive biology in the cancer^21^. Expression profiles representing proliferation had a more complex association with stage. Aurora kinase A expression increased, matching our expectations that more advanced cancer tends to have a higher level of proliferative signaling because it is innately more aggressive or has progressed to a more aggressive, genetically unstable state^5^. Another marker of proliferation, the “hallmark” of spermatogenesis, representing the “stem-like” activation of cancer cells to promote proliferation^22^, showed an upward trend that was slightly exaggerated by the difference in sample type. One interpretation might be that proliferation was more likely to be driven by alternative growth factor pathways.

Our findings are relevant to the current approach to management of metastatic and other locally advanced breast cancers. As blockers of proliferation, CDK4/6 inhibitors, in addition to endocrine therapy with AI or Fulvestrant, represent the current standard of care as a first-line therapy in the metastatic setting. However, the lack of a specific biomarker for treatment selection and the toxic effect of their extended use remain challenges in clinical practice, especially in their integration into adjuvant treatment. Maintenance of CDK4/6 inhibitors throughout progression in order to overcome endocrine treatment resistance represents an emerging practice, as there is a reported benefit for disease control^23,24^. Diminishing endocrine sensitivity necessitates the adjustment of the endocrine backbone while continuing with CDK4/6 inhibitors. However, there was conflicting data on the efficacy and predictive value of an *ESR1* mutation alone and in combination with other known somatic gene alterations associated with CDK4/6 inhibitor exposure in this therapeutic strategy^25^. Despite new endocrine and targeted treatment options following CDK4/6 inhibitor usage, beyond first-line, chemotherapy remains a valid choice, especially for clinically-aggressive disease without any druggable target.

When looking at the broader tumor microenvironment, we observed that an immune signature representing overall immune presence in these immunologically cold HR+/HER2− tumors increased with advancing stage^26^. This conflicts with the finding that metastatic lesions demonstrate low immunogenicity because they include a larger representation of immune-avoidant clones; however, it should be noted that most of our patients were not from Stage IV^26^. A growing, infiltrating, sustained cancer will evade anti-cancer immune signaling and activation^27^. Although statistically significant, the increase in immune cell gene expression with advancing stage was subtle, with little change from Stage III to IV. Therefore, the association that is observed could be due to less-differentiated tumors generally having slightly more cellular immune infiltration. Nevertheless, immune checkpoint inhibitors have not demonstrated efficacy in an HR+ setting.

At the chromosomal level, genomic instability signaling increased across stages, perhaps because mutations were more prevalent in higher-stage disease. This fits with an understanding of how cancer reflects an ever-destabilizing genome^28^. However, it is important to mention that we do not have DNA sequence or cytogenetic data, so we can only extrapolate about this possible effect.

Metabolic dysregulation is another important factor that drives changes. As cancer progresses, its energetic demands also increase^5^. The cancer biology signature heme metabolism, which describes the metabolic shift towards the overproduction of heme intermediates like porphyrin for sustaining the cancer^29^, was significantly higher in Stage IV in our study. It was also the only so-called “hallmark” transcriptional signature to progressively rise within Stage IV cancers based on the number of progression events. In cancer, there is a non-homeostatic upregulation of heme intermediates, called porphyrins, to fuel the cancer in a process called porphyrin overdrive^29^ Porphyrin overdrive stands out from other metabolic phenotypes like the Warburg Effect, glutamine addiction, and increased fatty acid anabolism because it represents the cancer-specific accumulation of heme intermediates^29^. Cancer cells rely on these intermediates for essential cellular processes, such as proliferation and the protection against iron-induced ferroptosis^30^. Imbalanced heme metabolism is a targetable metabolic phenotype because it is not present in healthy cells and is essential to the survival of the cancer^29^, so our finding invites further study for treatment options to exploit this phenotype. The site of metastasis did not appear to be associated with the level of heme metabolism gene expression signature in the biopsy samples (Supplementary Fig. S2). Other signatures that represented metabolism, but did not change significantly, were fatty acid metabolism, glycolysis, and oxidative phosphorylation (Table S2). However, other important metabolic phenotypes of cancer are not well represented by gene expression signatures, so we cannot speculate on their stage-related trends. The glycolytic state did not vary in mRNA expression, but this is not the most suitable measure anyway; metabolism occurs at the protein and biochemical level, so DNA or RNA expression profiles are only remotely related and cannot definitively measure this. Indeed, our samples could not take into account downstream enzymatic activity, so it is possible that even though we see changes at the transcriptional level, enzymatic activity may not change. Glycolytic state is a very important parameter of treatment resistance, even relevant to immunotherapy. A highly glycolytic state creates an unfavorable tumor microenvironment because it increases acidity, which in turn increases the activity of immune-suppressive T-cells^31^. This concept invites new clinical strategies to select patients who may particularly benefit from immunotherapy.

Overall, it is important to note that some signatures in our study contribute to the development of cancer, while others promote the progression of cancer to higher stages. Similarly, for some signatures, the differences in expression between cytology and tissue can be explained by the natural features of those signatures. For example, we would expect that CIN70 and spermatogenesis showed higher expression in the cytology at every stage because the chromosomal instability measure would be best captured within the more concentrated overall cellularity of an FNA, rather than the tissue sample, which would include stroma too. There was a similar confounding effect of sample type with tissue, as it has mesenchymal stroma, whereas cytology has very little^10^, so signatures like angiogenesis and epithelial-mesenchymal transition were much higher in tissue than in cytology.

Here, we would like to address the limitations of our study. To start, our study, taking place over many years, used a mature data set from samples that were freshly collected into RNA later and evaluated during that time by Affymetrix gene expression microarray technology, which predates contemporary RNA sequencing. Still, these signatures of interest are published as accurate when using these microarrays, and thus, we can make reasonable inferences^32^. We also acknowledge that these were not longitudinal samples capturing progression within a patient’s cancer timeline. Rather, each one came from a different patient. Studies of paired primary and subsequent metastasis provide more information about the individual’s molecular progression, but do not address the diversity across stages of primary disease^11,33^. Another limitation is that when assessing gene expression as a function of stage, as expected, there were fewer cytology samples from early stages and fewer tissue samples from later stages. This sample bias was a result of the context and method in which prospective samples can reasonably be obtained in prospective research protocols. Nevertheless, we feel their exact number and placement at these “boundary stages” is less important than the overall message of the trend (i.e., whether they go up or down or remain flat, when they change, and how quickly). Additionally, there were very few 1st-recurrence patients in our Stage IV-specific trends, because it is a difficult time to discuss with the patient, collecting optional research samples when the diagnosis has not been established at the time of biopsy. In that clinical setting, we would need to have relied on archival fixed biopsy samples, and those should be prioritized for clinical molecular testing. On the other hand, an advantage of our methodology was that our samples were all from fresh samples and unaffected by preanalytical effects of cold ischemia, fixation, or storage^34–36^. Moreover, for our Stage IV analysis, we were not able to take into account how long the interval was from prior treatment before they had the biopsy, or whether treatment was ongoing. It would also have been helpful to know the *PIK3CA* mutation status when evaluating the related gene expression signatures in the samples from metastases. The gene expression signatures and the cancer biology signatures are not validated diagnostic signatures, so technical variance may be an issue. Also, they are approximations of biology, not the same as studying biological function. This is true for most of the gene expression signatures that we analyzed. Still, our results provide insights that would guide more specific research.

In conclusion, our study summarizes the most significant transcriptional changes from high-quality clinical research biopsies that are related to the advancing stage across a large population of patients with HR+/HER2− breast cancer. We evaluated their biological associations with contemporary treatments for this disease and identified a few novel transcriptional changes with potential for novel therapeutic approaches.

## Methods

The cohort for these analyses was from the compilation of gene expression data from past studies wherein fresh tumor samples of HR+/HER2− breast cancer were profiled on U133A microarrays using uniform methodology. All patients gave informed written consent to take part in the study and for the use of tissue material for research purposes. The definition of HR+/HER2− breast cancer was based on the results of clinical biomarker testing, as reported at the institution where the sample was collected. HR-positive status was defined as >1% tumor nuclei stained positive for ER or PR by immunohistochemistry.

### Sample cohorts

Samples from primary surgical resection specimens at MDACC were collected fresh at the time of intraoperative specimen assessment by a dedicated breast pathologist (WFS) under research IRB protocols with MD Anderson Institutional Review Board (IRB) approval: LAB04-0093, LAB08-0823, LAB08-0824, and 2011-0007. A shave of the tissue from one side of the cut tumor mass was assessed by imprint cytology (to confirm malignancy) and diced in a petri dish and stored frozen at -80°C in *RNAlater* until use. A subset of fresh tumor samples collected at MDACC was included in the cohort with matched cytology and tissue samples^8^. For those, a cytologic sample was collected before the tissue sample. The freshly cut surface of the tumor was gently scraped using a small scalpel blade, a portion of that liquid sample was placed on a glass slide for assessment of the cytologic smear (to confirm malignancy), and the remainder was mixed into *RNAlater* solution. This was repeated 2–3 times with the liquid sample entirely added to the *RNAlater* solution. Immediately thereafter, the subjacent tumor tissue was shaved, diced, and added to a separate vial of *RNAlater* solution, as described above. Frozen tumor samples from collaborating institutions were stored frozen at -80°C and shipped on dry ice, as previously reported^7^.

Samples from needle biopsies, either FNA cytology or core biopsy tissue, were prospectively collected and immediately placed in *RNAlater* solution and held at room temperature at least 30 min to allow penetration of the preservative, then stored frozen at −80 °C in *RNAlater* until use, as previously described (approved IRB protocols LAB99-402, LAB04-0093, 2011-0007)^7,9^. We categorized AJCC Stage into groups of Stage I, IIA, IIB, III, and IV. If the sample was obtained from Stage I-III breast cancer prior to neoadjuvant treatment, then we used clinical Stage; otherwise, we used pathologic Stage if surgery was the primary treatment. For patients with documented Stage IV (i.e., documented distant metastasis), we further classified them into de novo (i.e., Stage IV at first presentation), 1st or 2nd metastatic relapse, or 3rd or later metastatic relapse.

### Gene expression profiling for target and reference transcripts

RNA was extracted, processed, and hybridized to Affymetrix human genome U133A microarrays (U133A GeneChip, Affymetrix, Santa Clara, CA, USA) as described previously. In brief, the raw intensity files were processed using the MAS5.0 algorithm to generate probe set-level intensities, normalized to a median array intensity of 600, log2-transformed^7,9^.

### Selection of genes and signatures

RNA was purified and hybridized to Affymetrix U133A microarrays (U133A GeneChip; Affymetrix, Santa Clara, CA) as reported previously^7,9^. Briefly, raw data were processed using the MAS5 algorithm as implemented in the affy3 package to generate probe-level intensities, scaled to a median intensity of 600, and log2-transformed. The data was then scaled to 1322 breast cancer reference genes within each sample following MAS5 normalization^7,9^.

As single genes, we selected the four most relevant genes for the classification of breast cancer, including the transcripts for estrogen receptor, progesterone receptor, and HER2 that represent the standard molecular therapeutic targets in breast cancer^37^. Research versions of the EndoPredict and Oncotype DX signatures, of the PAM50 and SCMGENE classifiers, the GGI and PIK3CA signatures, and gene modules were calculated using the genefu5 package^32^. The SET Index was calculated according to the original publication^8^. We obtained the cancer “Hallmarks” signatures from the Gene Set Enrichment Analysis Human MSigDB Collection.

In the matched pairs, the tissue samples were profiled at three different laboratories, and the average of those triplicate results was used to compare with the cytology profile that was performed at MDACC, as previously reported^8^.

### Statistical methods

Pearson’s correlation coefficient was used to compare the cross-tissue concordance correlation coefficient between the matched pairs of tissue and cytology samples for each transcript and signature. To evaluate the effect of disease stage on the genomic signatures, we fit linear regression models fitted to median values (MASS package7) and adjusted for the type of sample (cytology vs. tissue) as a covariate. *P* values were obtained by comparing the gradient of the regression line with zero (*t*-test). The same method was used to evaluate linear regression according to the number of recurrence events in the Stage IV cohort. All statistical analyses and computations were performed in R v. 4.5.1 and Bioconductor^38,39^.

## Supplementary information

Supplementary Information

## Data Availability

The microarray and accompanying data are located in NCBI GEO database under accession GSE326778.
