# Supplementary material for: Stage-related transcriptional changes in HR-positive/HER2-negative breast cancer
Source: NPJ Breast Cancer. 2026 Jul 2;12:140. doi: 10.1038/s41523-026-00973-6 (PMC13691097; doi:10.1038/s41523-026-00973-6)
Supplement: Supplementary file 1 [file 41523_2026_973_MOESM1_ESM.pdf]

SUPPLEMENTARY TABLES & FIGURES

Symmans W.S., *et al*. Stage-related transcriptional changes in HR-positive/HER2-negative breast cancer.

Contents.

Supplementary Figure S1. REMARK Diagram

Supplementary Table S1. Transcriptional Expression by Function of Cancer Biology

Supplementary Figure S2. Heme Metabolism Expression by Site of Metastasis

Supplementary Figure S1. REMARK Diagram

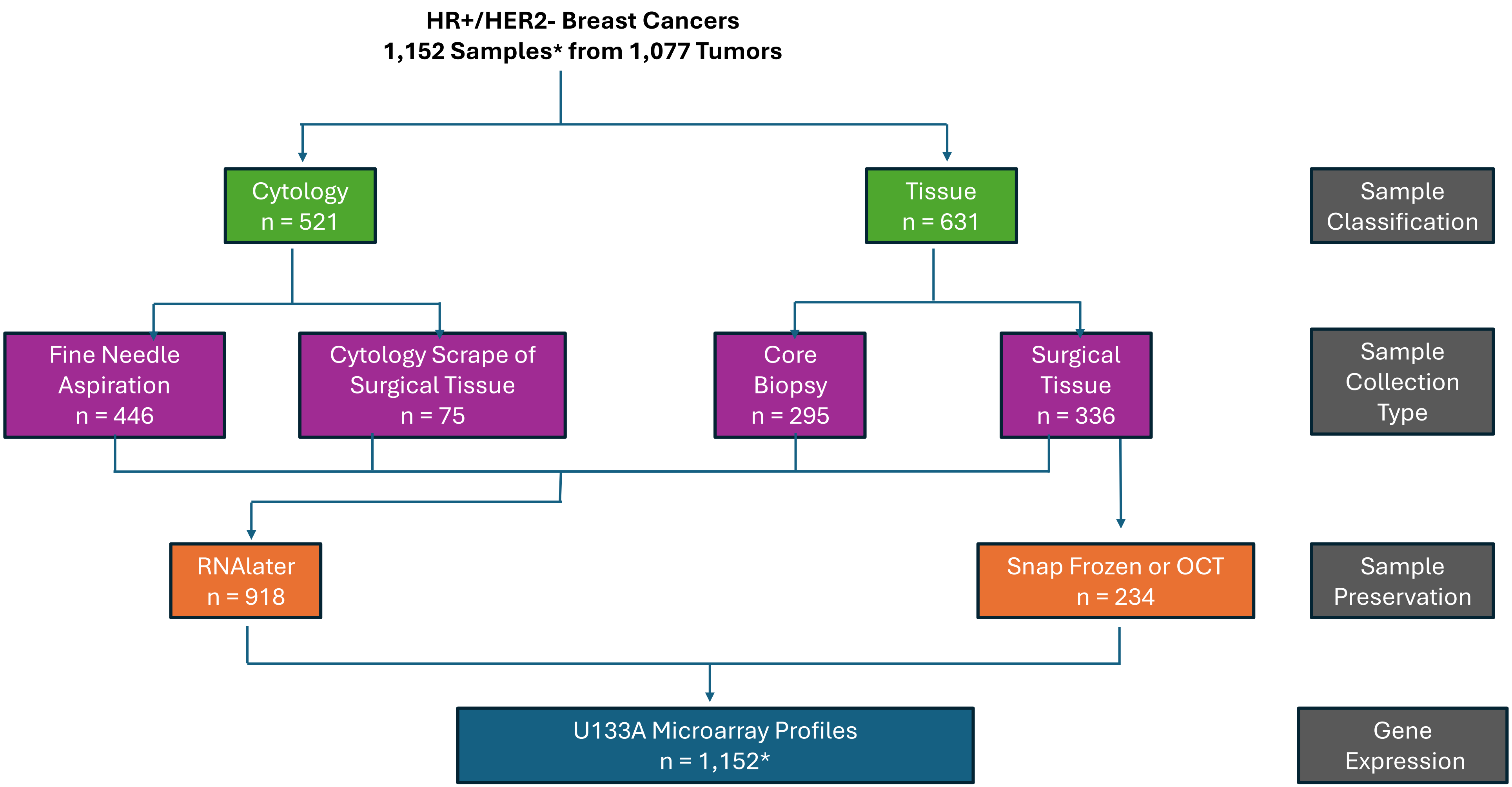

Samples collected from 1,077 unique tumors. *, 75 samples had matched pairs of cytology scrape and surgical tissue collected in RNAlater at time of surgery, per protocol, resulting in duplicate tumor profiling for comparison of gene expression concordance between sample collection type.

Supplementary Table S1: All Other Signatures

	Sample Matched Pairs			Clinical Samples Cohort					
	Cytology vs Tissue			Cytology vs Tissue			AJCC Stage Effect		
**Signature**	**Estimate**	**95% CI**	**p-value**	**Estimate**	**95% CI**	**p-value**	**Estimate**	**95% CI**	**p-value**
**Endocrine Activity**									
HALLMARK_ANDROGEN_RESPONSE	-0.081	-0.126, -0.035	**0.001**	-0.203	-0.232, -0.175	**< 0.001**	-0.007	-0.018, 0.003	0.157
HALLMARK_ESTROGEN_RESPONSE_EARLY	0.011	-0.054, 0.076	0.734	-0.130	-0.160, -0.100	**< 0.001**	-0.036	-0.046, -0.025	**< 0.001**
HALLMARK_ESTROGEN_RESPONSE_LATE	0.010	-0.047, 0.066	0.733	-0.108	-0.135, -0.080	**< 0.001**	-0.023	-0.033, -0.013	**< 0.001**
**Cell Signaling**									
BetaCatenin Signature	-0.016	-0.041, 0.009	0.196	-0.036	-0.050, -0.023	**< 0.001**	-0.010	-0.014, -0.005	**< 0.001**
HALLMARK_APICAL_JUNCTION	-0.155	-0.201, -0.109	**< 0.001**	-0.208	-0.227, -0.188	**< 0.001**	-0.003	-0.010, 0.005	0.479
HALLMARK_APICAL_SURFACE	-0.172	-0.228, -0.115	**< 0.001**	-0.093	-0.120, -0.066	**< 0.001**	-0.004	-0.013, 0.006	0.455
HALLMARK_HEDGEHOG_SIGNALING	-0.127	-0.179, -0.075	**< 0.001**	-0.102	-0.127, -0.077	**< 0.001**	-0.011	-0.020, -0.002	0.019
HALLMARK_IL2_STAT5_SIGNALING	-0.198	-0.245, -0.151	**< 0.001**	-0.181	-0.203, -0.158	**< 0.001**	0.011	0.003, 0.019	**0.006**
HALLMARK_IL6_JAK_STAT3_SIGNALING	-0.186	-0.256, -0.116	**< 0.001**	-0.071	-0.102, -0.039	**< 0.001**	0.012	0.001, 0.024	0.036
HALLMARK_KRAS_SIGNALING_DN	0.053	0.018, 0.088	**0.003**	0.174	0.152, 0.197	**< 0.001**	0.006	-0.003, 0.014	0.172
HALLMARK_KRAS_SIGNALING_UP	-0.293	-0.341, -0.244	**< 0.001**	-0.163	-0.187, -0.138	**< 0.001**	0.005	-0.003, 0.014	0.226
HALLMARK_MTORC1_SIGNALING	0.054	-0.025, 0.133	0.182	-0.082	-0.121, -0.042	**< 0.001**	0.021	0.007, 0.036	**0.003**
HALLMARK_NOTCH_SIGNALING	-0.077	-0.133, -0.020	**0.008**	-0.243	-0.270, -0.216	**< 0.001**	-0.008	-0.018, 0.001	0.095
HALLMARK_PI3K_AKT_MTOR_SIGNALING	0.014	-0.033, 0.062	0.559	-0.022	-0.044, -0.000	0.045	0.014	0.006, 0.022	**0.001**
HALLMARK_TGF_BETA_SIGNALING	-0.186	-0.250, -0.122	**< 0.001**	-0.335	-0.364, -0.307	**< 0.001**	-0.006	-0.016, 0.004	0.267

HALLMARK_TNFA_SIGNALING_VIA_NFKB	-0.202	-0.268, -0.135	**< 0.001**	-0.223	-0.251, -0.195	**< 0.001**	-0.006	-0.016, 0.004	0.241
HALLMARK_WNT_BETA_CATENIN_SIGNALING	-0.029	-0.084, 0.026	0.298	-0.089	-0.112, -0.065	**< 0.001**	-0.002	-0.010, 0.007	0.728
PIK3cags	0.006	-0.049, 0.061	0.838	-0.067	-0.089, -0.046	**< 0.001**	-0.010	-0.018, -0.003	**0.009**
SRC Signature	-0.021	-0.040, -0.001	0.041	-0.022	-0.033, -0.012	**< 0.001**	-0.009	-0.013, -0.005	**< 0.001**
STAT1 Signature	-0.160	-0.265, -0.054	**0.003**	0.017	-0.022, 0.057	0.391	0.016	0.001, 0.030	0.034
**Proliferation and Grade**									
E2F3 Signature	0.018	0.001, 0.035	0.043	0.011	0.003, 0.020	**0.008**	-0.005	-0.008, -0.002	**0.001**
GENIUS	0.339	0.180, 0.497	**< 0.001**	0.330	0.269, 0.391	**< 0.001**	0.032	0.009, 0.054	**0.006**
GGI	0.094	0.004, 0.184	0.042	0.132	0.097, 0.167	**< 0.001**	0.033	0.020, 0.045	**< 0.001**
HALLMARK_E2F_TARGETS	0.149	0.038, 0.260	**0.009**	0.017	-0.029, 0.063	0.472	0.030	0.014, 0.047	**< 0.001**
HALLMARK_G2M_CHECKPOINT	0.131	0.040, 0.222	**0.005**	0.001	-0.038, 0.040	0.969	0.027	0.013, 0.041	**< 0.001**
HALLMARK_MYC_TARGETS_V1	0.150	0.045, 0.254	**0.005**	-0.099	-0.150, -0.049	**< 0.001**	0.021	0.002, 0.039	0.027
HALLMARK_MYC_TARGETS_V2	0.170	0.072, 0.267	**0.001**	-0.026	-0.071, 0.018	0.241	0.013	-0.003, 0.029	0.116
MAPK Signature	-0.019	-0.032, -0.006	**0.004**	0.023	0.015, 0.031	**< 0.001**	-0.002	-0.005, 0.001	0.251
Ras Signature	-0.061	-0.084, -0.037	**< 0.001**	-0.046	-0.058, -0.034	**< 0.001**	-0.004	-0.008, 0.000	0.071
**Metabolism and Cellular Activity**									
HALLMARK_ADIPOGENESIS	-0.066	-0.118, -0.013	0.015	-0.203	-0.236, -0.170	**< 0.001**	-0.008	-0.020, 0.004	0.167
HALLMARK_BILE_ACID_METABOLISM	-0.033	-0.072, 0.006	0.095	0.021	0.002, 0.041	0.033	-0.007	-0.014, 0.000	0.063
HALLMARK_CHOLESTEROL_HOMEOSTASIS	0.071	0.005, 0.136	0.034	-0.092	-0.128, -0.057	**< 0.001**	-0.000	-0.013, 0.012	0.944
HALLMARK_FATTY_ACID_METABOLISM	0.016	-0.040, 0.073	0.562	-0.095	-0.126, -0.064	**< 0.001**	-0.005	-0.016, 0.007	0.427
HALLMARK_GLYCOLYSIS	-0.004	-0.053, 0.044	0.861	-0.117	-0.143, -0.091	**< 0.001**	0.002	-0.008, 0.011	0.747
HALLMARK_OXIDATIVE_PHOSPHORYLATION	0.117	0.027, 0.207	0.011	-0.138	-0.188, -0.089	**< 0.001**	-0.001	-0.019, 0.017	0.877

HALLMARK_PANCREAS_BETA_CELLS	-0.058	-0.109, -0.007	0.026	0.078	0.053, 0.102	**< 0.001**	0.010	0.001, 0.019	0.034
HALLMARK_PEROXISOME	0.059	0.012, 0.106	0.014	-0.030	-0.055, -0.004	0.021	-0.003	-0.012, 0.006	0.526
HALLMARK_PROTEIN_SECRETION	-0.034	-0.102, 0.034	0.324	-0.156	-0.192, -0.119	**< 0.001**	0.003	-0.010, 0.017	0.615
**Genome Instability and Mutagenesis**									
CIN70 Signature	0.126	0.036, 0.216	**0.006**	0.111	0.075, 0.148	**< 0.001**	0.025	0.012, 0.038	**< 0.001**
HALLMARK_DNA_REPAIR	0.115	0.048, 0.183	**0.001**	-0.068	-0.102, -0.033	**< 0.001**	0.008	-0.004, 0.021	0.200
HALLMARK_MITOTIC_SPINDLE	0.006	-0.045, 0.058	0.814	-0.122	-0.147, -0.098	**< 0.001**	0.008	-0.000, 0.017	0.063
HALLMARK_REACTIVE_OXYGEN_SPECIES_PATHWAY	-0.012	-0.083, 0.060	0.748	-0.138	-0.174, -0.102	**< 0.001**	0.008	-0.005, 0.021	0.246
HALLMARK_SPERMATOGENESIS	0.063	0.021, 0.105	**0.003**	0.182	0.162, 0.202	**< 0.001**	0.014	0.007, 0.021	**< 0.001**
HALLMARK_UV_RESPONSE_DN	-0.248	-0.303, -0.192	**< 0.001**	-0.351	-0.374, -0.329	**< 0.001**	-0.019	-0.028, -0.011	**< 0.001**
HALLMARK_UV_RESPONSE_UP	0.008	-0.041, 0.058	0.741	-0.092	-0.117, -0.067	**< 0.001**	0.010	0.001, 0.019	0.035
**Resisting Cell Death**									
CASP3 Signature	-0.013	-0.048, 0.023	0.478	0.071	0.051, 0.091	**< 0.001**	0.004	-0.003, 0.011	0.274
HALLMARK_APOPTOSIS	-0.191	-0.234, -0.149	**< 0.001**	-0.238	-0.261, -0.216	**< 0.001**	-0.003	-0.011, 0.006	0.525
HALLMARK_P53_PATHWAY	-0.088	-0.128, -0.048	**< 0.001**	-0.189	-0.210, -0.168	**< 0.001**	-0.008	-0.015, -0.000	0.049
HALLMARK_UNFOLDED_PROTEIN_RESPONSE	0.099	0.032, 0.166	**0.004**	-0.157	-0.194, -0.120	**< 0.001**	0.003	-0.011, 0.016	0.691
HALLMARK_XENOBIOTIC_METABOLISM	-0.071	-0.110, -0.033	**< 0.001**	-0.091	-0.112, -0.071	**< 0.001**	-0.002	-0.009, 0.006	0.648
TAMR13	2.248	1.626, 2.871	**< 0.001**	2.180	1.919, 2.440	**< 0.001**	0.223	0.128, 0.318	**< 0.001**
**Immune Activity**									
HALLMARK_ALLOGRAFT_REJECTION	-0.218	-0.301, -0.134	**< 0.001**	-0.058	-0.093, -0.022	**0.001**	0.016	0.003, 0.029	0.014
HALLMARK_COMPLEMENT	-0.224	-0.286, -0.163	**< 0.001**	-0.159	-0.187, -0.131	**< 0.001**	0.015	0.004, 0.025	**0.005**
HALLMARK_INTERFERON_ALPHA_RESPONSE	-0.134	-0.254, -0.014	0.028	-0.163	-0.216, -0.110	**< 0.001**	0.027	0.008, 0.047	**0.005**

HALLMARK_INTERFERON_GAMMA_RESPONSE	-0.201	-0.300, -0.102	**< 0.001**	-0.183	-0.225, -0.140	**< 0.001**	0.028	0.012, 0.043	**0.001**
IMMUNE Signature	-1459	-1946, -971	**< 0.001**	93	-150, 336	0.452	146	56, 234	**0.001**
**Invasion and Microenvironment**									
HALLMARK_ANGIOGENESIS	-0.395	-0.460, -0.330	**< 0.001**	-0.433	-0.462, -0.404	**< 0.001**	-0.003	-0.013, 0.008	0.614
HALLMARK_COAGULATION	-0.275	-0.322, -0.228	**< 0.001**	-0.256	-0.279, -0.233	**< 0.001**	-0.006	-0.014, 0.002	0.166
HALLMARK_EPITHELIAL_MESENCHYMAL_TRANSITION	-0.473	-0.550, -0.396	**< 0.001**	-0.569	-0.602, -0.536	**< 0.001**	-0.016	-0.028, -0.004	0.011
HALLMARK_HYPOXIA	-0.184	-0.231, -0.137	**< 0.001**	-0.241	-0.263, -0.218	**< 0.001**	-0.001	-0.009, 0.007	0.770
HALLMARK_INFLAMMATORY_RESPONSE	-0.210	-0.275, -0.144	**< 0.001**	-0.056	-0.085, -0.027	**< 0.001**	0.011	0.001, 0.022	0.036
HALLMARK_MYOGENESIS	-0.169	-0.218, -0.119	**< 0.001**	-0.159	-0.181, -0.137	**< 0.001**	-0.000	-0.008, 0.008	0.931
PLAU Signature	-0.311	-0.373, -0.250	**< 0.001**	-0.343	-0.371, -0.315	**< 0.001**	-0.009	-0.019, 0.001	0.090
STROMA Signature	-2486	-2819, -2152	**< 0.001**	-2221	-2391, -2052	**< 0.001**	-60	-120, 4	0.066
VEGF Signature	0.126	0.086, 0.167	**< 0.001**	-0.010	-0.031, 0.011	0.338	-0.006	-0.014, 0.002	0.119

These cancer biology signatures from the 50 listed in the Broad Institute Gene Set Enrichment Analysis were included in this Supplementary Table S1 if they: 1) had stage effect with significance level $p \geq 0.01$, 2) were redundant with a different biological signature that was already presented in Table 1 of the manuscript, or 3) demonstrated substantial difference in measurements from matched pairs of tissue and cytology samples. For the clinical samples cohort, this meant that the gradient of the regression line across staging was not significantly up or down. The “estimate” column quantifies the difference in means between cytology and tissue for matched pair samples, with a positive value indicating that the cytology was higher than tissue. “Estimate” in the clinical cohort is the slope of the regression line with increasing stage category as an integer.

Supplementary Figure S2. Heme Metabolism Expression by Site of Metastasis

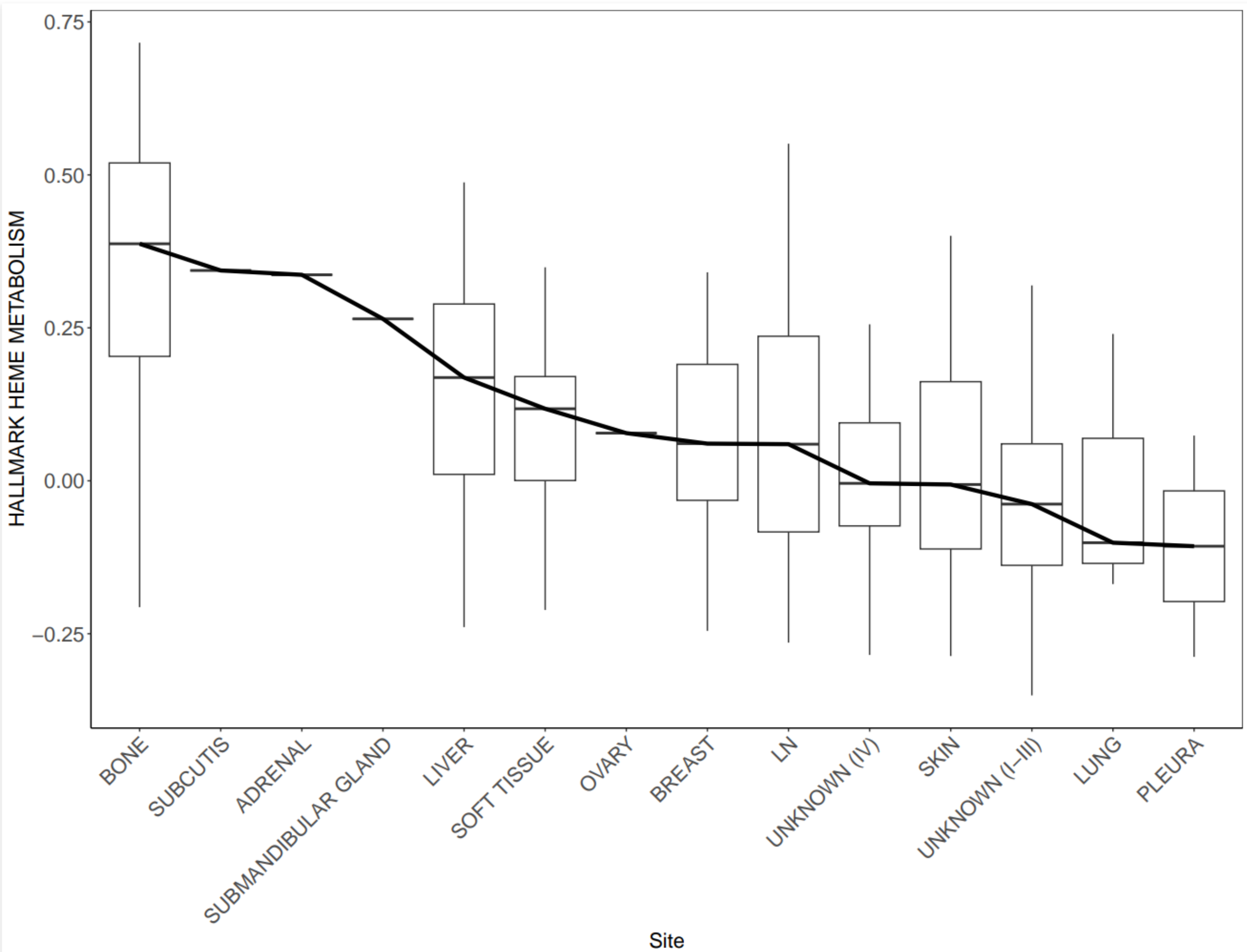

Box plots showing mean expression values and interquartile range of heme metabolism in different tissues. The black line connects the mean values from all samples.
